# Development of a global urban greenness indicator dataset for 1,000+ cities

**DOI:** 10.1016/j.dib.2023.109140

**Published:** 2023-04-11

**Authors:** Jennifer D. Stowell, Catherine Ngo, Marcia Pescador Jimenez, Patrick L. Kinney, Peter James

**Affiliations:** a Department of Environmental Health, Boston University School of Public Health, 715 Albany Street, Boston, MA 02118, United States; b Consultant, Boston University School of Public Health, 715 Albany Street, Boston, MA 02118, United States; c Department of Epidemiology, Boston University School of Public Health, Boston, MA, 715 Albany Street, Boston, MA 02118, United States; d Department of Population Medicine, Harvard Medical School and Harvard Pilgrim Health Care Institute, Boston, MA, Landmark Center, 401 Park Dr #401, Boston, MA 02215, United States; e Department of Environmental Health, Harvard T. Chan School of Public Health, 677 Huntington Avenue, Boston, MA 02115, United States

**Keywords:** Climate change, Built environment, Tree canopy, Vegetation

## Abstract

Global climate change has sparked efforts to adapt to increasing temperatures, especially in urban areas that experience increased day and nighttime temperatures due to the urban heat island effect. The addition of greenspace has been suggested as a possible means for urban centers to respond to increasing urban temperatures. Thus, it is important for urban planning and policymakers to have access to data on greenspace specific at a fine spatial resolution. This dataset consists of information on peak and annual average 1 × 1 km Normalized Difference Vegetation Index (NDVI) for over 1,000 global urban centers, which is an objective satellite-based measure of vegetation. Population-weighted values for both peak and annual average NDVI and include an indicator of greenness, with seven levels ranging from extremely low to extremely high are provided. Additional information regarding the climate zone (using the Köppen-Geiger climate classification) and level of development (using the Human Development Index or HDI) for each city is included. Analyses were repeated in 2010, 2015, and 2020 to provide the ability to track urban greenness over time. Data are provided in tabular format with summaries presented in both tables and graphics. These data can be used to inform policy and planning and can be used as an indicator for a variety of climate and health investigations.

**Specifications Table**

**Table:** 

Subject	Environmental Science: Global and Planetary Change
Specific subject area	Processed remote sensing for global urban greenness classification.
Type of data	Tables Figures Charts Maps Graph
How the data were acquired	Publicly available data were acquired using a combination of satellite imagery, global gridded population, human settlement data, climate regions, and perimeters of global urban areas. Data integration was accomplished using Google Earth Engine (GEE) and R statistical software. Using Landsat 7, 8, and 9, processed images were used to calculate NDVI and utilized the “ee.Algorrithms.Landsat.simpleComposite()”, “reduceRegions”, and “ee.Reducer.mean” methods in GEE. Outputs from GEE were used to calculate various city-specific Normalized Difference Vegetation Index (NDVI) measures in R.
Data format	Raw Data: .csv file containing processed data for each city Analyzed & Filtered Data: .tiff graphical data and tabular data
Description of data collection	Extracted NDVI data were used to generate data points for each city, including measures of peak NDVI and annual mean 1 × 1 km NDVI, collected every 16 days. Additional measures are included representing population-weighted values for both peak and annual mean NDVI. Cities were selected based on population size (500,000 or more). In countries without urban areas of this size, the largest urban area was included. Only cities with data for all years (2010, 2015, 2020) were included in the final dataset. Cities were grouped by Greenness Indicator, Human Development Index (HDI), and climate region for additional analysis.
Data source location	• Institution: Boston University School of Public Health • City: Boston, Massachusetts • Country: United States
Data accessibility	Data used for the generation of the current dataset were acquired by the following: 1. Landsat 7, 8, and 9 data provided by NASA/USGS and can be accessed via: https://earthexplorer.usgs.gov/ 2. Population data are provided by NASA's Socioeconomic Data and Applications Center (SEDAC) hosted by CEISIN at Columbia University: https://sedac.ciesin.columbia.edu/data/collection/gpw-v4 3. Urban spatial extents are available from the Global Human Settlement Urban center Database R2019A: https://ghsl.jrc.ec.europa.eu/ghs_stat_ucdb2015mt_r2019a.php
	4. Human development index classifications are provided by the United Nations Human Development Report Office: https://hdr.undp.org/data-center/human-development-index#/indicies/HDI 5. Climate regions from the Köppen-Geiger climate classification system are available at: http://glass.umd.edu/KGClim/ The Global Urban Greenness Indicator data is publicly available and be accessed at the following location: Repository name: Harvard Dataverse Data identification number: https://doi.org/10.7910/DVN/TMWYHB Direct URL to data: https://doi.org/10.7910/DVN/TMWYHB
Related research article	M. Romanello, C. Di Napoli, P. Drummond, C. Green, H. Kennard, P. Lampard, et al. The 2022 report of the Lancet Countdown on health and climate change: health at the mercy of fossil fuels. Lancet. Volume 400 (2022), Issue 10,363, P1619–1654. https://doi.org/10.1016/S0140–6736(22)01,540–9

## Value of the Data

• These data are a useful tool in determining urban centers that suffer from lack of green space
• Policymakers and city planners may find this dataset useful in aiding decision-making and climate-related analyses of urban centers (i.e., developing plans to mitigate urban heat islands).
• Population health researchers can apply this dataset to multiple investigations, including reducing temperatures in urban centers, increasing physical activity in urban centers, and the use of greenspace to improve health outcomes
• This dataset is made readily available in a universal format that is both streamlined and accessible to individuals regardless of expertise or experience in the underlying data or platforms.
• Data may be used as inputs for urban research models to study a variety of health- and non-health-related subjects.

## 1 Objective

While the Lancet Reports provide a summary of our key findings and methods, they lack details on processes used to generate the raw data [1], [2], [3]. This paper elaborates on the application of cloud computing technology to conduct large-scale analyses of remote sensing data, with details on the data sources, functions and parameters, and analytical metrics used. This report includes additional analyses on urban greenness stratified by climate region and human development index. This paper gives details regarding the data, methods, and analysis for creating an urban greenness indicator.

## 2 Data Description

The “Global Greenspace Indicator Dataset” consists of three types of data, including processed data, tabular data, and graphical data [4]. The raw data was processed from each data source (see below) and compiled the results in the processed data file. The processed data are provided in ‘.csv’ format and include information on urban areas, multiple measures of the Normalized Vegetation Index (NDVI), levels of development utilizing the Human Development Index (HDI), and categories of the greenness indicator. Additional summaries and visualizations of the data are included as tables (‘.csv’) and graphics (‘.tiff’).

**Fig. 1:**
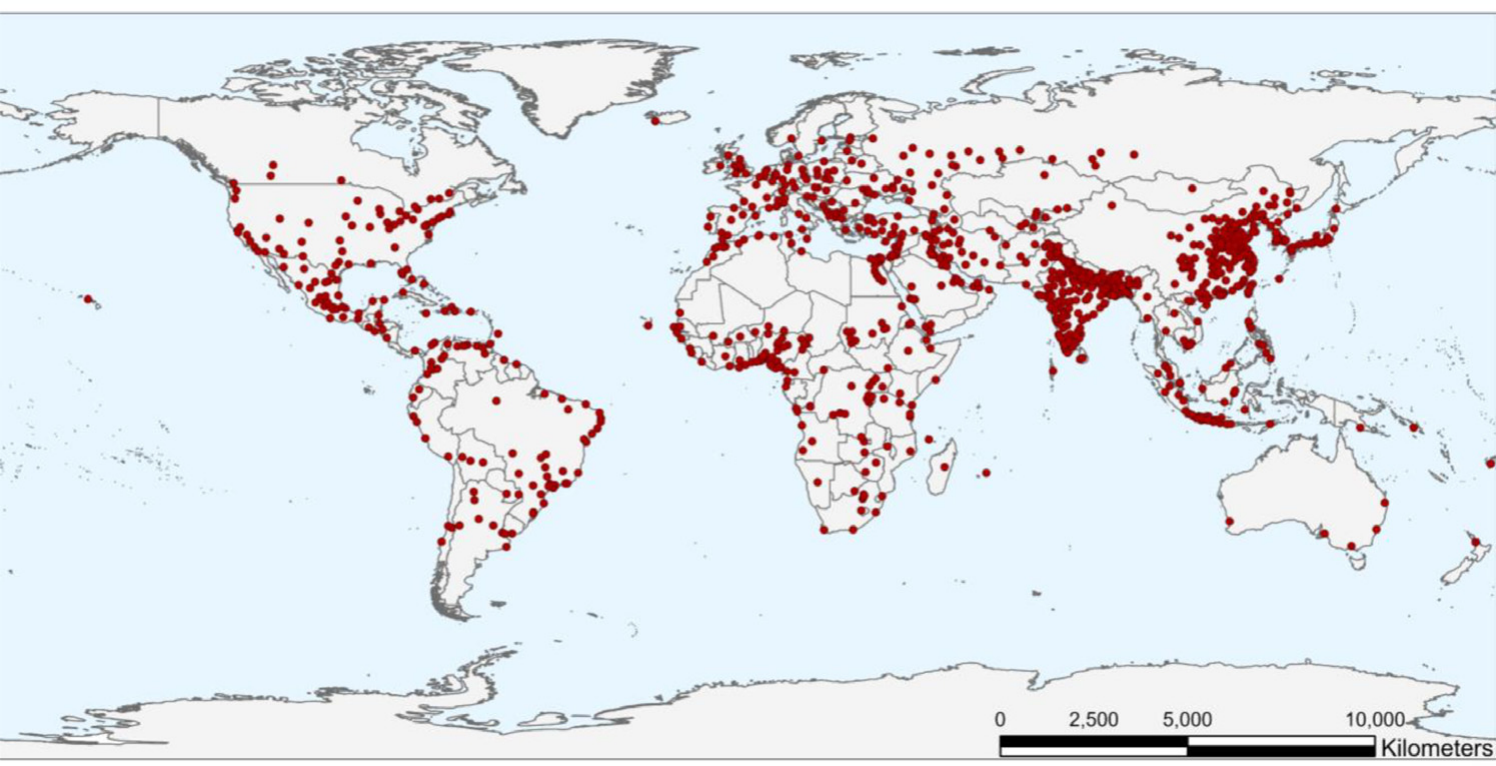
Distribution of cities included in the greenness indicator.

**Fig. 2:**
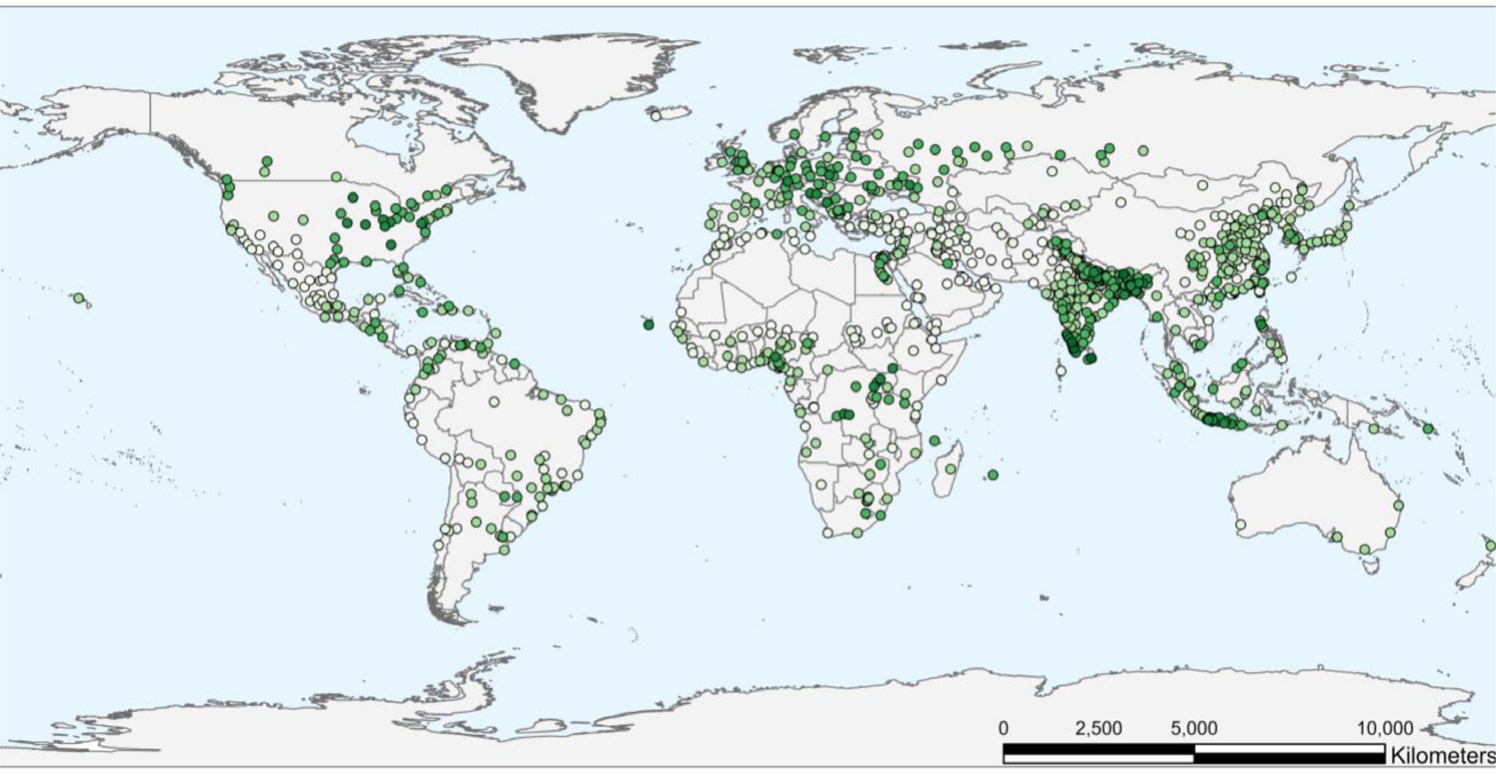
Annual population-weighted peak urban greenness by NDVI level.

**Fig. 3:**
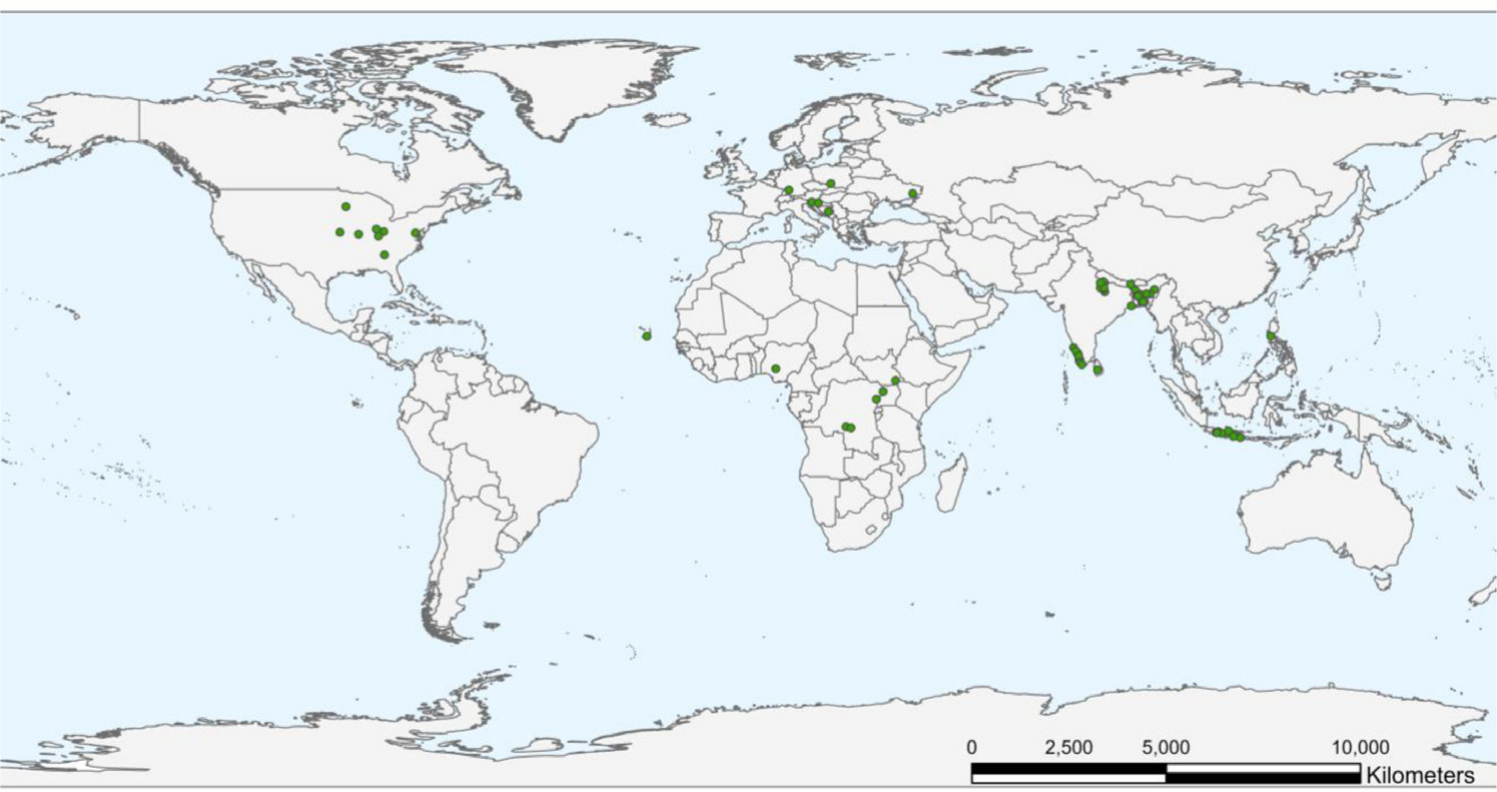
Distribution of cities with a high or very high level of greenness based on its population-weighted peak NDVI in 2020.

**Fig. 4:**
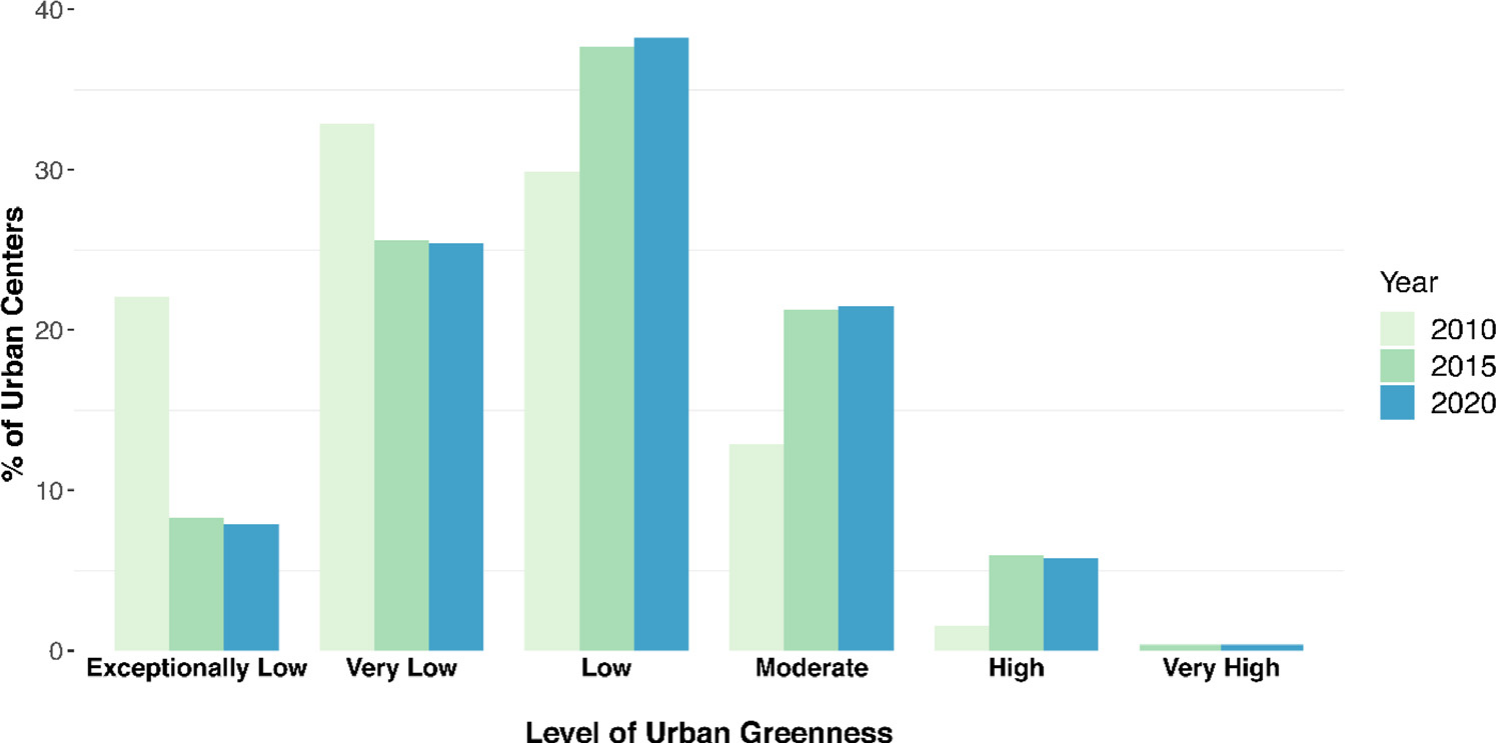
Proportion and count within each NDVI category by year, based on population-weighted peak average NDVI.

**Fig. 5:**
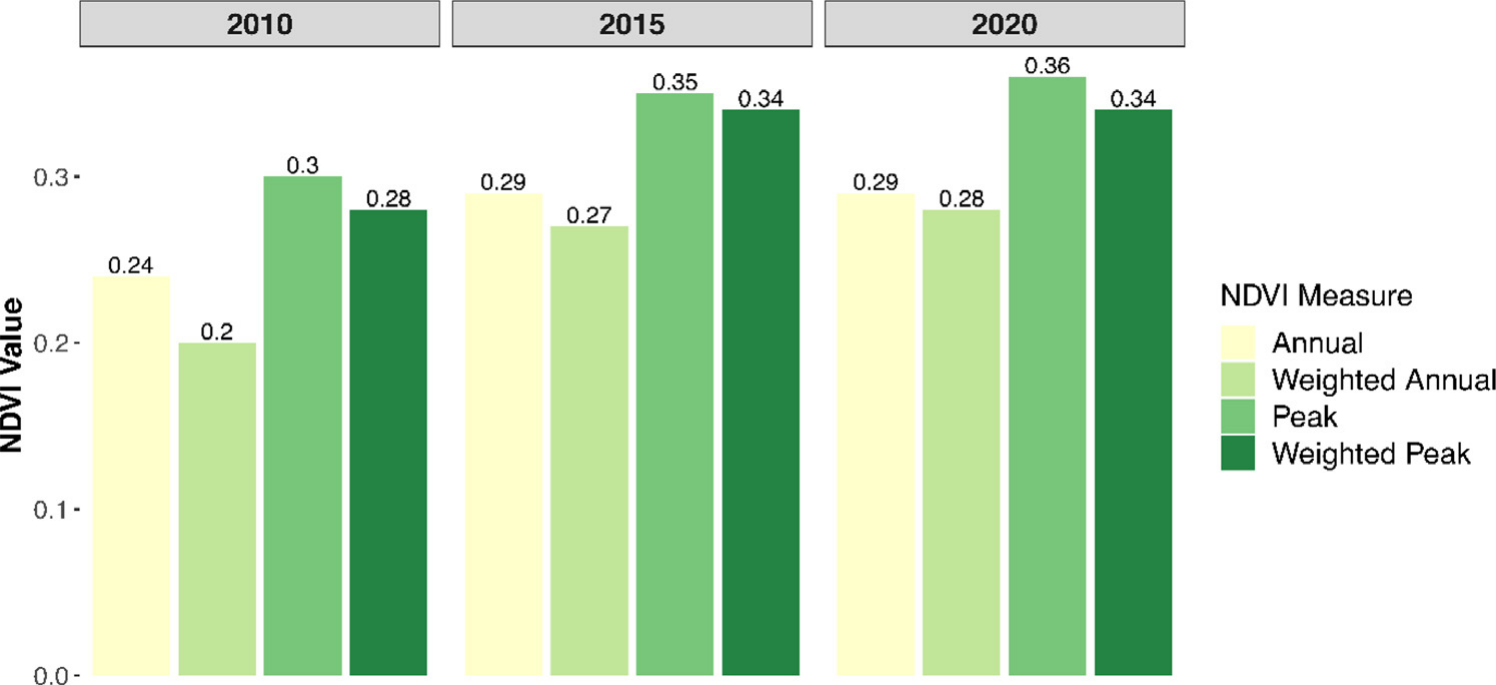
Weighted and unweighted peak and annual NDVI averages for all cities and all years.

Fig. 1 presents the global distribution of included urban centers in each included country. Fig. 2 presents the population-weighted annual peak NDVI for each of the included urban areas, indicated by the color of each point. Information in Table 1 summarizes the NDVI measures for each level of the greenness indicator. Fig. 3 provides the global distribution of the greenest cities for 2020 (high or very high greenness levels). Fig. 4 charts the change over time in population-weighted peak levels between 2010 and 2020, shown as percentages of the total number of urban areas for each of the greenness levels. For each year, mean unweighted and weighted peak and annual NDVI values were calculated and displayed in Fig. 5.

**Table 1:** Measures of NDVI by greenness indicator level for 1038 global urban areas.

2020				
Greenness Indicator	Peak NDVI	Annual NDVI	Pop-weighted Peak NDVI	Pop-weighted Annual NDVI
Exceptionally Low	0.16	0.14	0.15	0.13
Very Low	0.28	0.23	0.26	0.21
Low	0.36	0.30	0.35	0.28
Moderate	0.45	0.36	0.44	0.35
High	0.53	0.43	0.53	0.44
Very High	0.61	0.53	0.6	0.53

2015				

Exceptionally Low	0.16	0.14	0.15	0.13
Very Low	0.28	0.23	0.26	0.21
Low	0.36	0.3	0.35	0.28
Moderate	0.44	0.35	0.44	0.35
High	0.53	0.43	0.53	0.43
Very High	0.63	0.54	0.62	0.54

2010				

Exceptionally Low	0.18	0.14	0.15	0.1
Very Low	0.28	0.22	0.25	0.18
Low	0.36	0.28	0.34	0.23
Moderate	0.45	0.34	0.44	0.28
High	0.53	0.42	0.53	0.36
Very High	NA	NA	NA	NA

**Fig. 6:**
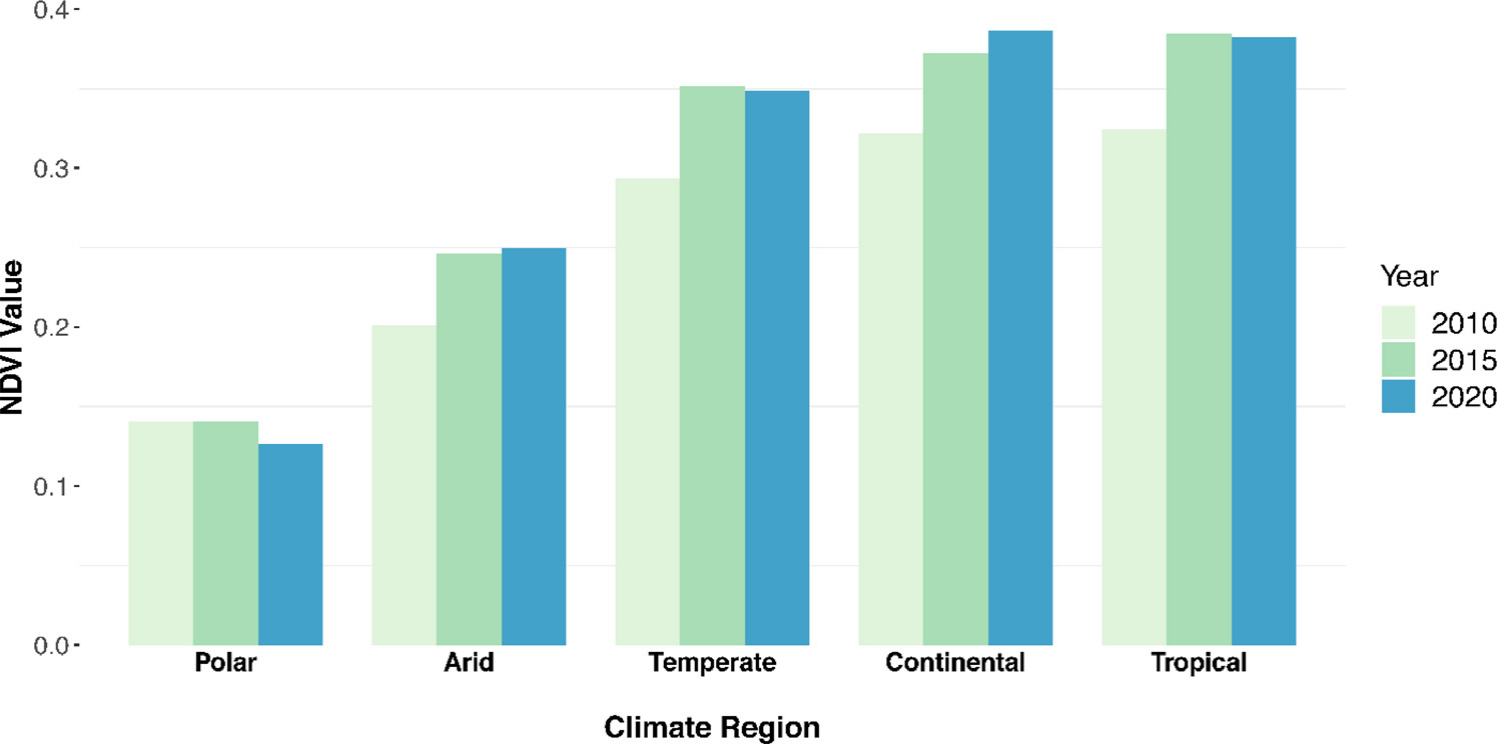
Temporal changes in the mean population-weighted peak NDVI for cities in each climate region in 2010, 2015, and 2020.

**Fig. 7:**
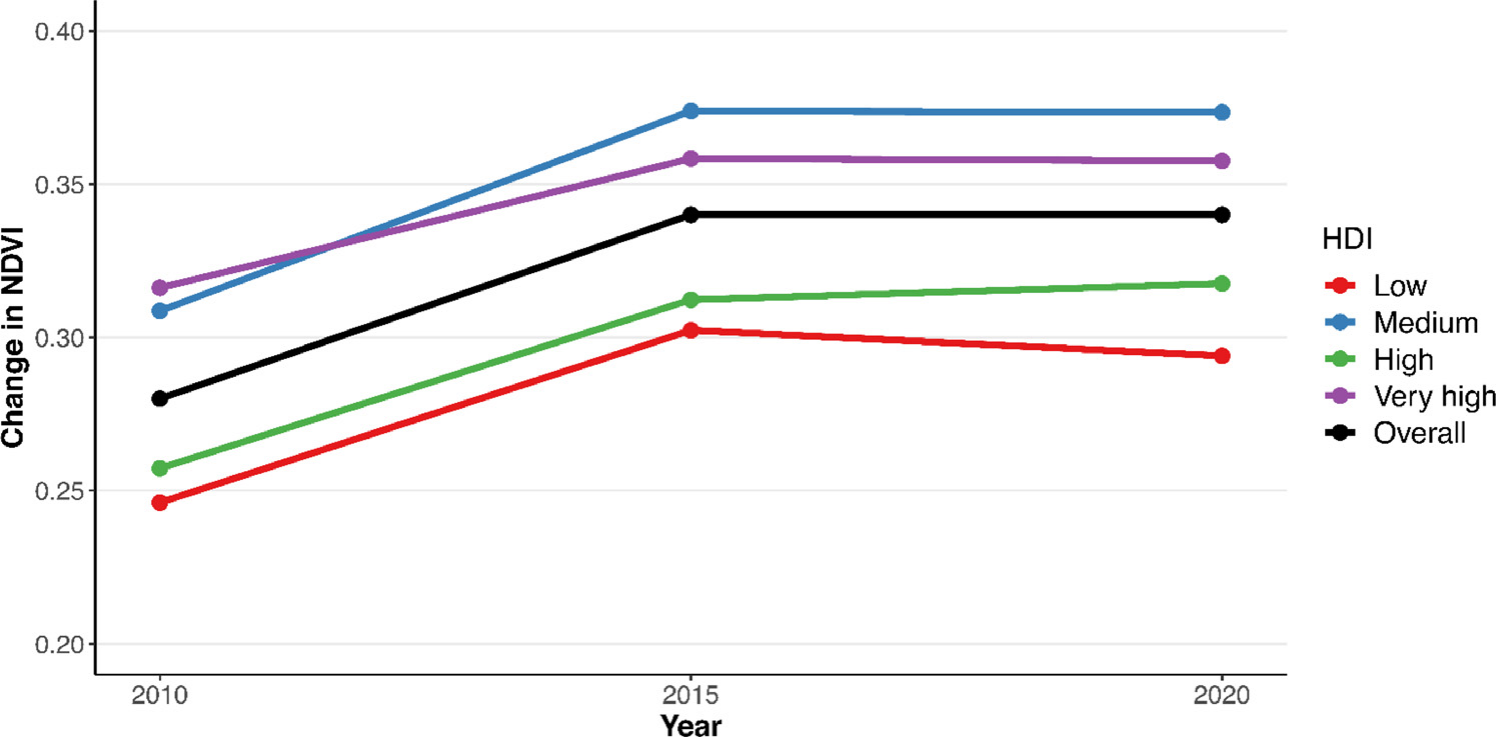
Temporal changes in the mean population-weighted peak NDVI for cities by HDI category in 2010, 2015, and 2020.

The dataset is summarized by specific subcategories that may be of interest to end users, including climate region and level of development. Fig. 6 charts the mean population-weighted peak NDVI for cities over time and information in Table 2 summarizes the NDVI measures by HDI category. After classifying each urban area by the Köppen-Geiger Climate Classification category, the average population-weighted peak NDVI is calculated for each of the five categories: polar, arid, temperate, continental, and tropical (as seen in Fig. 7) and mean population-weighted peak NDVI is summarized in Table 3.

**Table 2:** Measures of NDVI by HDI level for 1038 global urban areas.

2020				
HDI Level	Peak NDVI	Annual NDVI	Pop-weighted Peak NDVI	Pop-weighted Annual NDVI
Low	0.31	0.25	0.29	0.23
Medium	0.38	0.32	0.37	0.31
High	0.34	0.28	0.32	0.25
Very High	0.36	0.29	0.36	0.28

2015				

Low	0.32	0.26	0.30	0.24
Medium	0.38	0.31	0.37	0.31
High	0.34	0.28	0.31	0.25
Very High	0.37	0.29	0.36	0.28

2010				

Low	0.27	0.21	0.25	0.17
Medium	0.32	0.24	0.31	0.20
High	0.29	0.22	0.26	0.19
Very High	0.33	0.26	0.32	0.22

**Table 3:** Measures of NDVI by climate region for 1038 global urban areas.

2020				
Climate Region	Peak NDVI	Annual NDVI	Pop-weighted Peak NDVI	Pop-weighted Annual NDVI
Polar	0.15	0.13	0.13	0.11
Arid	0.26	0.21	0.25	0.21
Temperate	0.36	0.30	0.35	0.29
Continental	0.39	0.27	0.38	0.26
Tropical	0.40	0.34	0.38	0.32

2015				

Polar	0.17	0.14	0.14	0.12
Arid	0.26	0.21	0.25	0.20
Temperate	0.37	0.30	0.35	0.28
Continental	0.38	0.26	0.37	0.25
Tropical	0.40	0.34	0.38	0.32

2010				

Polar	0.15	0.12	0.14	0.11
Arid	0.22	0.17	0.20	0.14
Temperate	0.31	0.24	0.29	0.21
Continental	0.33	0.23	0.32	0.19
Tropical	0.36	0.29	0.32	0.22

## 3 Experimental Design, Materials and Methods

Data on population size and density for 2010, 2015 and 2020 were collected from the Gridded Population of the World, Version 4 (GPWv4): Population Density, Revision 11 at Columbia University's Center for International Earth Science Information Network (CIESIN) [5]. Urban area spatial extents were taken from the Global Human Settlement Urban center Database R2019A (GHS) [6]. Urban centers with populations larger than 500,000 were included in the analysis. For countries lacking urban areas with populations greater than 500,000, the most populated urban area in the country was selected for inclusion. Based on these methods, 1042 major urban centers across 174 countries were identified. However, remote sensing data were unavailable (either due to cloud cover or equipment malfunction) for two of the countries for all seasons, which resulted in our final dataset comprised of 1038 urban centers across 172 countries.

The Human Development Index (HDI) classifications for each country were provided by the United Nations Human Development Report Office (UN HDRO) [7]. In addition, climate regions were defined using an updated version of the Köppen-Geiger climate classification system at 1 km spatial resolution [8]. For this study, the five general classes of climate were used, which include tropical, arid, temperate, cold and polar. Stratification using this system allows for increased understanding and tracking changes in greenness by region over time.

NDVI was derived from the joint National Aeronautics and Space Administration (NASA)/U.S. Geological Survey (USGS) Landsat program [9]. Landsat 8 images were used to calculate NDVI in 2015 and 2020 and Landsat 7 for 2010. While we could have used Landsat 7 images to calculate NDVI for all years, a hardware equipment failure in 2003 resulted in large amounts of missing data since then. As a result, Landsat 8 images were used, when possible, to minimize data gaps.

ArcGIS Pro 3.0.4 was utilized to subset the GHS layer by cities with populations of at least 500,000 [10]. Shapefiles of these cities were then imported into Google Earth Engine (GEE). In GEE, remotely sensed data was downloaded for each year and applied the ee.Algorithms.Landsat.simpleComposite() method and the default parameters to create a Landsat composite for each season. GEE's built-in function to compute NDVI was then applied. With this approach, all non-cloud-contaminated satellite images collected during our specified time periods were used in averaging the NDVI for each season and city. Seasons were defined based on northern hemisphere seasonal parameters: December 1 to February 31 as “winter”, March 1 to May 31 as “spring”, June 1 to August 31 as “summer”, and September 1 to November 30 as “fall”. The same dates were used to denote southern hemisphere seasons but reversed to reflect known seasonal patterns: June 1 to August 31 as “winter”, September 1 to November 30 as “spring”, December 1 to February 31 as “summer”, and March 1 to May 31 as “fall”. Any negative NDVI values, indicating water, were set to zero. Finally, mean NDVI for each city and season were determined using the reduceRegions method. The function reduceRegions was applied over the cloud-free Landsat composite per season, reduced them over the shapefile of GHS cities, and set the reducer to ee.Reducer.mean, and the scale to 100 m. This was repeated using the same method for each year to generate output including the mean NDVI values for each season and city in the years 2010, 2015, and 2020.

NDVI values above 0 were classified the level of greenness into 7 indicator levels as outlined in Table 4, indicating NDVI values at exceptionally low, very low, low, moderate, high, very high, and exceptionally high levels. Using GEE, population-weighted NDVI for each season and year was calculated to assess population exposure to green space within each urban center. The equation used for the population-weighted NDVI is shown below:

$$\frac{\sum_{i=1}^{n}\left(NDVI_{i}*population_{i}\right)}{\sum_{i=1}^{n}population_{i}}$$

where i is an individual pixel, and n is the total number of pixels within an urban center. Using the cloud-free Landsat composites with negative NDVI values set to zero, each NDVI value was multiplied by the population size (from CIESIN/GPWv4) of the corresponding year within the same 1 × 1 km pixel. Applying reduceRegions, the numerator (i.e., sum of the weighted values) was generated with the same parameters as above, except the reducer was changed to ee.Reducer.sum. For the denominator (i.e., sum of the weights), images were loaded from “CIESIN/GPWV4/population-density/2010 or 2015 or 2020.” The reduceRegions command was applied to the population density layer using the same parameters as the numerator. The sum of the weighted values and the sum of the weights were determined for each urban area and all seasons per year using GEE. We did not calculate the final population-weighted NDVI in GEE. The remainder of the analysis was done using R Statistical Software (R) to compute variations of the population-weighted averages, as well as to process and analyze data [11].

**Table 4:** Greenness Indicator Levels and corresponding NDVI values.

Indicator Level	NDVI Value
Exceptionally high	NDVI ≥ 0.7
Very high	0.6 ≤ NDVI < 0.7
High	0.5 ≤ NDVI < 0.6
Moderate	0.4 ≤ NDVI < 0.5
Low	0.3 ≤ NDVI < 0.4
Very Low	0.2 ≤ NDVI < 0.3
Exceptionally Low	NDVI < 0.2

Outputs in GEE were generated for each urban area in the dataset, including NDVI by season per city per year, population-weighted NDVI by season per city per year, and population of each city. Using R, four metrics were calculated per year per city based on the GEE outputs, including peak NDVI (maximum NDVI across the four seasons), annual mean based on the four-season average NDVI (annual), population-weighted peak NDVI (pop-weighted peak), and population-weighted annual mean NDVI (pop-weighted annual).

While our GEE script selected only images with minimal cloud cover, remote sensing data can have missing information for several reasons. [12,13] As a result, some cities were missing NDVI data for one or more seasons. Other missingness reflected a known equipment failure on Landsat 7 (used for 2010 values, yielding 303 missing values in 2010. Using Landsat 8, there were 16 and 21 missing values in 2015 and 2020, respectively. Our R script removed those missing values and calculated the metrics described above based on available data. Future iterations of the data will include additional years and we will consider the addition of urban blue space to form a hybrid indicator.

## Ethics Statement

The authors have read and followed the ethical requirements for publication in Data in Brief. This dataset does not include human subjects, animal experiments, or data collected from social media platforms and is based solely on the use of secondary data from sources listed in this manuscript.

## Declaration of Competing Interest

The authors declare that they have no known competing financial interests or personal relationships that could have appeared to influence the work reported in this paper.

## Data Availability

Global Greenspace Indicator Dataset (Original data) (Dataverse).
